# Supplementary figures and images for: Transcriptome and Metabolome Provide Insights into Fruit Ripening of Cherry Tomato (Solanum lycopersicum var. cerasiforme)
Source: Plants (Basel). 2023 Oct 9;12(19):3505. doi: 10.3390/plants12193505 (PMC10575466; doi:10.3390/plants12193505)

a

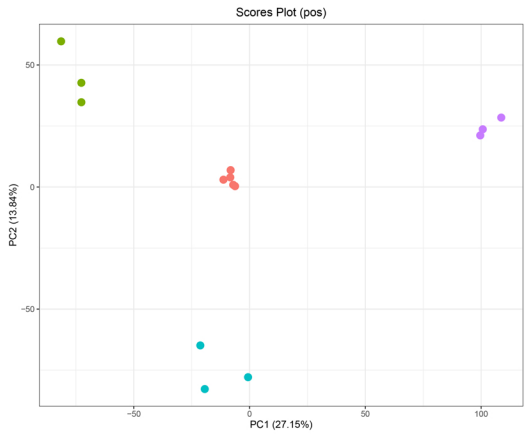

b

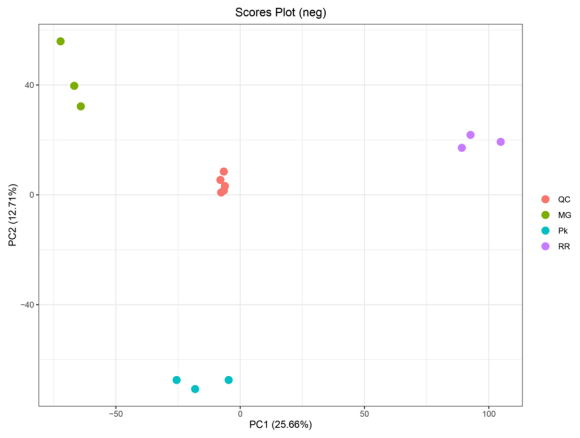

c

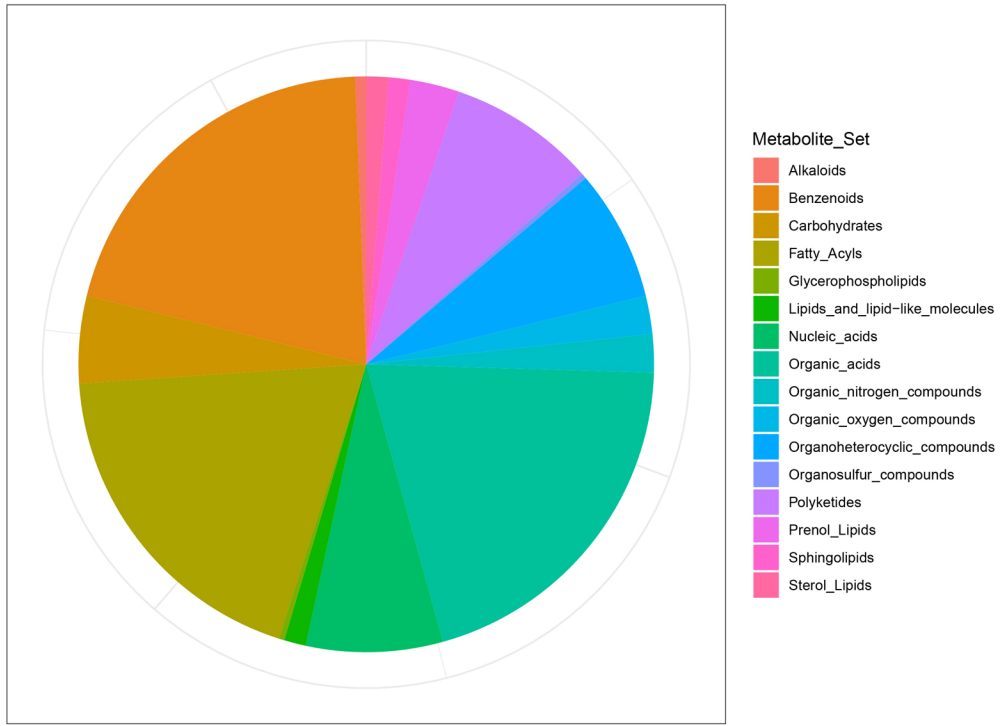

Supplement: Supplementary file 1 [file plants-12-03505-s001.zip › FigureS1.pdf]

a

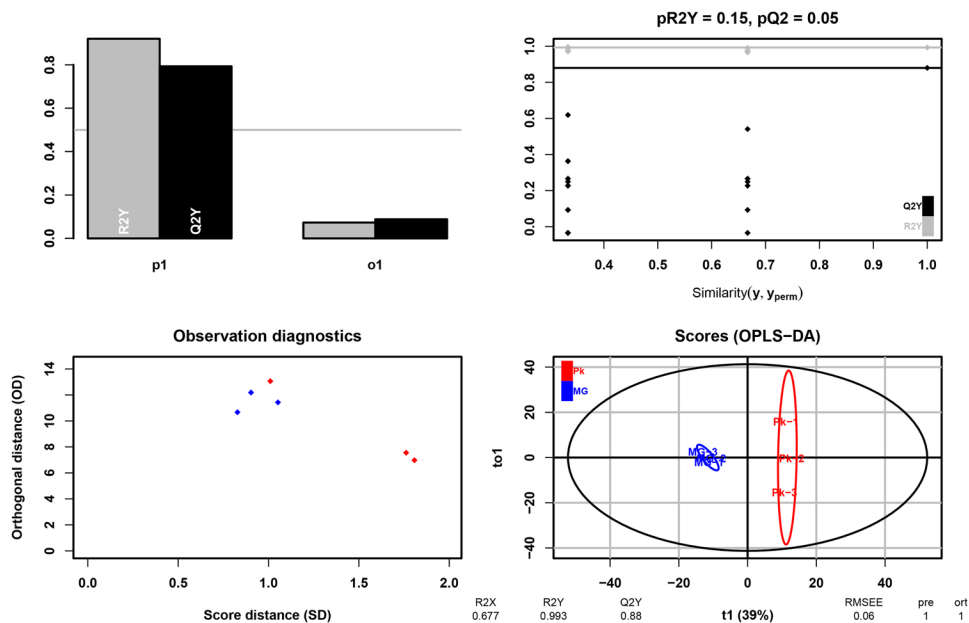

b

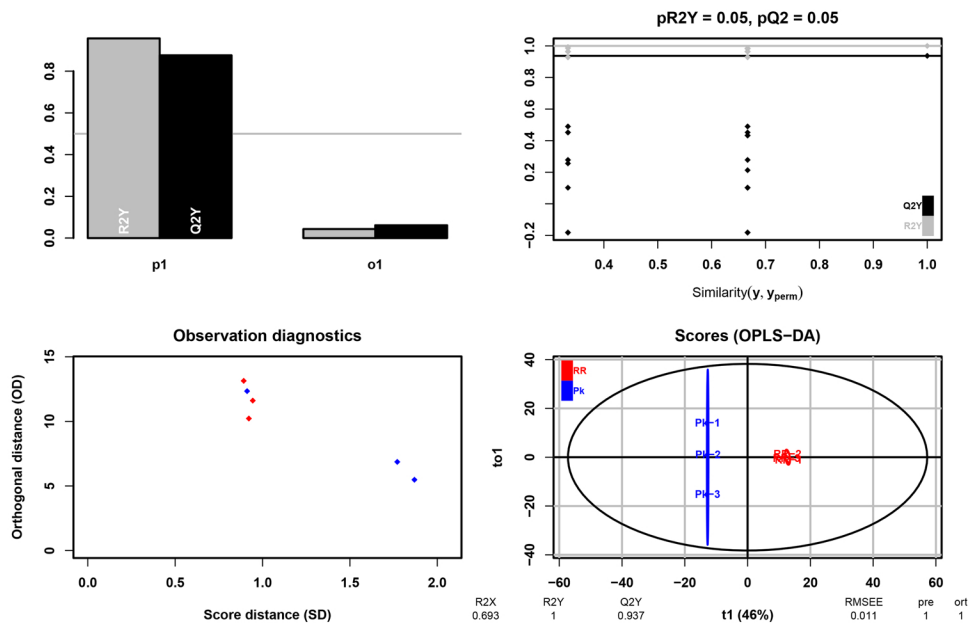

Supplement: Supplementary file 1 [file plants-12-03505-s001.zip › FigureS2.pdf]
